# Replicative Fitness of a SARS-CoV-2 20I/501Y.V1 Variant from Lineage B.1.1.7 in Human Reconstituted Bronchial Epithelium

**DOI:** 10.1128/mBio.00850-21

**Published:** 2021-07-06

**Authors:** Franck Touret, Léa Luciani, Cécile Baronti, Maxime Cochin, Jean-Sélim Driouich, Magali Gilles, Laurence Thirion, Antoine Nougairède, Xavier de Lamballerie

**Affiliations:** a Unité des Virus Émergents, Aix-Marseille University, IRD 190, Inserm 1207, Marseille, France; Icahn School of Medicine at Mount Sinai

**Keywords:** 20I/501Y.V1, B.1.1.7, SARS-CoV-2, *ex vivo*, *in vitro*, replicative fitness, variant

## Abstract

Since its emergence in 2019, circulating populations of the new coronavirus (CoV) continuously acquired genetic diversity. At the end of 2020, a variant named 20I/501Y.V1 (lineage B.1.1.7) emerged and replaced other circulating strains in several regions. This phenomenon has been poorly associated with biological evidence that this variant and the original strain exhibit different phenotypic characteristics. Here, we analyze the replication ability of this new variant in different cellular models using for comparison an ancestral D614G European strain (lineage B1). Results from comparative replication kinetics experiments *in vitro* and in a human reconstituted bronchial epithelium showed no difference. However, when both viruses were put in competition in human reconstituted bronchial epithelium, the 20I/501Y.V1 variant outcompeted the ancestral strain. All together, these findings demonstrate that this new variant replicates more efficiently and may contribute to a better understanding of the progressive replacement of circulating strains by the severe acute respiratory CoV-2 (SARS-CoV-2) 20I/501Y.V1 variant.

## OBSERVATION

Novel severe acute respiratory syndrome coronavirus 2 (SARS-Cov-2) emerged in China by the end of 2019 and rapidly spread worldwide. In a few months, the D614G spike mutation was rapidly fixed in almost all circulating SARS-CoV-2 populations, without evidence of higher CoV disease 2019 (COVID-19) mortality or clinical severity (1). It is still being debated whether it is due to a random founder effect (1) or, more probably, whether the mutation enhances viral loads in the upper respiratory tract, increasing the infectivity and stability of virions (2–4).

In September 2020, a variant named 20I/501Y.V1 from lineage B.1.1.7 (initially named VOC 2 2020212/01) emerged in the United Kingdom. It spread rapidly and is becoming dominant in Western Europe (5) and the United States (6). There is consistent epidemiological evidence that this so-called “UK variant” is more efficiently transmitted (7) than the preexisting European strains, in particular in young patients. Moreover, this variant has also been associated in some studies with an increased risk of mortality (8–10), without any differences in symptomatology (11).

Here, we present a comprehensive analysis of the replication ability *in vitro* and *ex vivo* of the 20I/501Y.V1 variant (strain UVE/SARS-CoV-2/2021/FR/7b isolated in February 2021 in Marseille, France; GISAID accession no. EPI_ISL_918165), using for comparison the lineage B.1 BavPat D614G strain that circulated in Europe in February/March of 2020.

**FIG 1:**
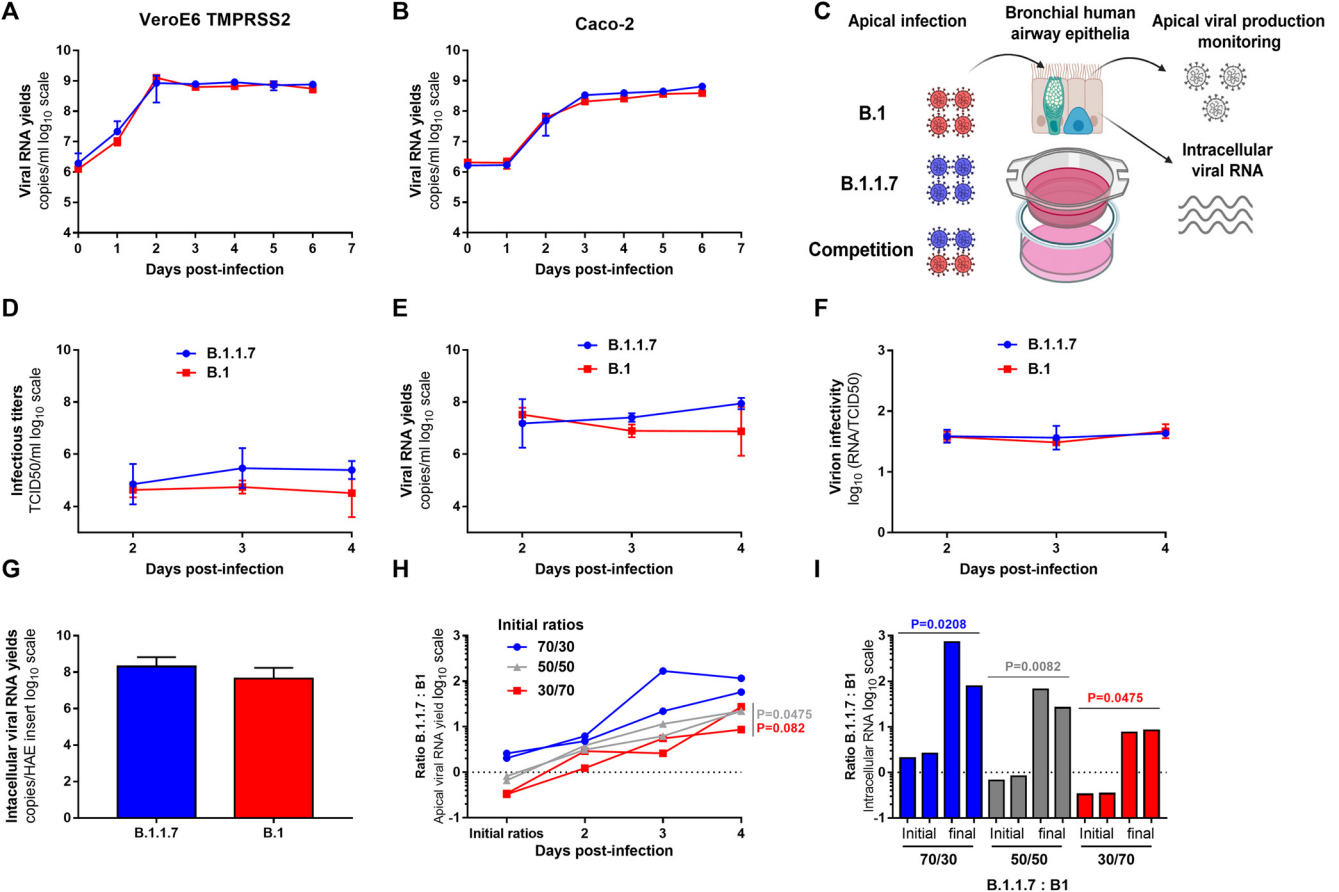
*In vitro* and *ex vivo* replication ability of a 20I/501Y.V1 (B.1.1.7) variant in comparison with a lineage B.1 D614G strain. (A and B) Replication kinetics in VeroE6 TMPRSS2 (A) and Caco-2 (B) cells. Viral replication was assessed using an RT-qPCR assay. (C) Graphical representation of experiments with reconstituted human airway epithelium (HAE) of bronchial origin. (D and E) Kinetics of virus excretion at the apical side of the epithelium measured using a 50% tissue culture infective dose (TCID_50_) assay (D) and an RT-qPCR assay (E). (F) Estimation of virion infectivities (i.e., the ratio of the number of infectious particles to the number of viral RNA particles). (G) Intracellular viral RNA yields measured at 4 dpi using an RT-qPCR assay. (A to G) Data represent means ± standard deviations (SD) from triplicate experiments. No statistical difference was observed between the two viral strains (*P* > 0.05, unpaired Mann-Whitney test). (H) Follow-up of the B.1.1.7/B.1 ratios at the apical side. Each line represents results from an HAE insert. (I) Individual B.1.1.7/B.1 ratios estimated from intracellular viral RNAs at 4 dpi (I). (H and I) *P* values were determined against the initial ratios using the Kruskal-Wallis test followed by an uncorrected Dunn *post hoc* analysis. The graphical representation was created with BioRender.

The first experiments were performed in two cell lines: VeroE6/TMPRSS2 cells, commonly used for SARS-Cov-2 isolation and propagation (12), and Caco-2 cells, which endogenously express the ACE2 receptor and TMPRSS2 coreceptor at levels similar to those in Calu-3 cells (13). Results of these experiments revealed highly similar replication kinetics, supporting the results of complete genome sequencing of both viral strains with regard to the integrity of the multibasic cleavage site in the spike protein (Fig. 1A and B and see Table S1 in the supplemental material) (14).

10.1128/mBio.00850-21.3 TABLE S1 Detailed single-nucleotide changes with a frequency of 20% or greater in the complete genome of the B.1.1.7 (20I/501Y.V1) stock (passage 2 [P2]). No single-nucleotide changes with a frequency of >20% were found in B.1 (BavPat1 D614G). S, synonymous; N.S, nonsynonymous. Download Table S1, DOCX file, 0.01 MB. Copyright © 2021 Touret et al. 2021 Touret et al. https://creativecommons.org/licenses/by/4.0/ This content is distributed under the terms of the Creative Commons Attribution 4.0 International license.

We then assessed the replicative fitness of both strains using a previously described model of reconstituted human airway epithelium (HAE) of bronchial origin (15). Following the inoculation of the epithelia through their apical side at a multiplicity of infection (MOI) of 0.1 in order to mimic the natural route of infection, we monitored the excretion of new virions at the apical side between 2 and 4 days postinfection (dpi) and measured the intracellular viral RNA yields at 4 dpi. Infectious titers (Fig. 1D) and viral RNA yields (Fig. 1E) at the apical side at 3 and 4 dpi, as well as intracellular viral RNA yields at 4 dpi (Fig. 1G), were slightly higher for the B.1.1.7 variant. However, differences were not significant, and estimated relative virion infectivities (i.e., the ratio of the number of infectious particles to the number of viral RNA particles) were similar for the two viruses at all sampling times (Fig. 1F). All together, these results are in line with our findings for common cell lines and with a recent report (16).

Based on these results, we performed competition experiments, which have previously been demonstrated to be effective to detect moderate replicative fitness differences (2, 17). Accordingly, we inoculated epithelia with both viruses simultaneously as described above, sampled the apical side between 2 and 4 dpi, and extracted intracellular viral RNA yields at 4 dpi. Three infection inoculum ratios (B.1.1.7/B1 ratios, 70/30, 50/50, and 30/70) were used. Using two specific reverse transcription-quantitative PCR (RT-qPCR) assays (Fig. S1), we estimated the proportion of each viral genome in the viral population (expressed as the B.1.1.7/B1 ratio in Fig. 1H and I). Regardless of the initial ratio, we observed similar patterns in which B1 was outcompeted by the B.1.1.7 variant; all B.1.1.7/B1 ratio values estimated from apical-side washes were above 1 and over 57, 22, and 8 at 4 dpi for epithelia inoculated at the initial ratios of 70/30, 50/50 and 30/70, respectively (Fig. 1H). Notably, B.1.1.7/B1 ratios measured at 4 dpi were significantly higher than the initial 50/50 and 30/70 inoculum ratios (*P* = 0.0475 and *P* = 0.0082, respectively, with the Kruskal-Wallis test with an uncorrected Dunn *post hoc* analysis). Similar results were observed when estimating the B.1.1.7/B1 ratios from intracellular viral RNAs (Fig. 1I); B.1.1.7/B1 ratios measured at 4 dpi were significantly higher than the initial 50/50 and 30/70 inoculum ratios (*P* = 0.0208, *P* = 0.0082, and *P* = 0.0475 with the 70/30, 50/50, and 30/70 inoculum ratios, respectively, as determined by the Kruskal-Wallis test with an uncorrected Dunn *post hoc* analysis).

10.1128/mBio.00850-21.2 FIG S1 Design and validation of two specific RT-qPCR systems for the BavPat D614G (B.1) and 20I/501Y.V1 (B.1.1.7) strains. (A) Detail of the hybridization positions of the common forward and reverse primers. The first nucleotide of the forward primer corresponds to nucleotide 11205 of the reference genome MW368440.1 (gene coding for NSP6). The probe exploiting a 9-nucleotide deletion in the genome of the 20I/501Y.V1 variant in the middle of the amplified sequence achieves the specificity of the system. (B) Validation of the systems using T7-generated synthetic RNA. Test of a specific BavPat system with a BavPat (1a) or 20I/501Y.V1 (1b) *in vitro* synthesized RNA (IVT). Test of a specific 20I/501Y.V1 system with a BavPat (2a) or 20I/501Y.V1 (2b) IVT. No cross-amplification was observed between the two systems. (C) Results using nucleic acid extracts from the HAE supernatant infected with the 20I/501Y.V1 variant or BavPat D614G virus. No cross-amplification was observed. Download FIG S1, DOCX file, 0.2 MB. Copyright © 2021 Touret et al. 2021 Touret et al. https://creativecommons.org/licenses/by/4.0/ This content is distributed under the terms of the Creative Commons Attribution 4.0 International license.

Our results demonstrated that the 20I/501Y.V1 (B.1.1.7) variant is more fit than the lineage B.1 BavPat D614G strain in reconstituted bronchial human epithelium. This may be explained by the presence of the N501Y mutation in the receptor binding domain (RBD) of the spike protein, which enhances viral particle binding to the ACE2 receptor (18). This may translate into a fitness advantage, as demonstrated in a recent study with engineered viral strains (19). Similar observations have been made with the D614G mutation, with which the new G614 strains overcame the original D614 strains when put in competition (2). All together, these findings may contribute to a better understanding of the progressive replacement of circulating strains by the SARS-CoV-2 20I/501Y.V1 variant (20).

10.1128/mBio.00850-21.1 TEXT S1 Supplemental materials and methods. Download Text S1, DOCX file, 0.02 MB. Copyright © 2021 Touret et al. 2021 Touret et al. https://creativecommons.org/licenses/by/4.0/ This content is distributed under the terms of the Creative Commons Attribution 4.0 International license.

10.1128/mBio.00850-21.4 DATA SET S1 Virus stock complete genome sequences. Download Data Set S1, DOCX file, 0.04 MB. Copyright © 2021 Touret et al. 2021 Touret et al. https://creativecommons.org/licenses/by/4.0/ This content is distributed under the terms of the Creative Commons Attribution 4.0 International license.
