# Supplementary material for: Replicative Fitness of a SARS-CoV-2 20I/501Y.V1 Variant from Lineage B.1.1.7 in Human Reconstituted Bronchial Epithelium
Source: mBio. 2021 Jul 6;12(4):e00850-21. doi: 10.1128/mBio.00850-21 (PMC8406299; doi:10.1128/mBio.00850-21)
Supplement: TABLE S1 [file mbio.00850-21-st001.docx]

**Supplemental Table 1**: Detailed single nucleotide changes with a frequency of 20% or greater in the complete genome of B.1.1.7 (20I/501Y.V1) stock (P2). No single nucleotide changes with a frequency >20% were found in B.1 (BavPat1 D614G). S: Synonymous, N.S: Non synonymous

| **Strain** | **Position** | **ORF** | **Nucleotide change** | **Mutation type** | **Frequency at P2** |
| --- | --- | --- | --- | --- | --- |
| **B .1.1.7** | **11391** | ORF1ab1 | C>T | N.S | 25% |
|  | **11687** | ORF1ab1 | T>C | N.S | 46% |
