# Supplementary material for: Replicative Fitness of a SARS-CoV-2 20I/501Y.V1 Variant from Lineage B.1.1.7 in Human Reconstituted Bronchial Epithelium
Source: mBio. 2021 Jul 6;12(4):e00850-21. doi: 10.1128/mBio.00850-21 (PMC8406299; doi:10.1128/mBio.00850-21)
Supplement: FIG S1 [file mbio.00850-21-sf001.docx]

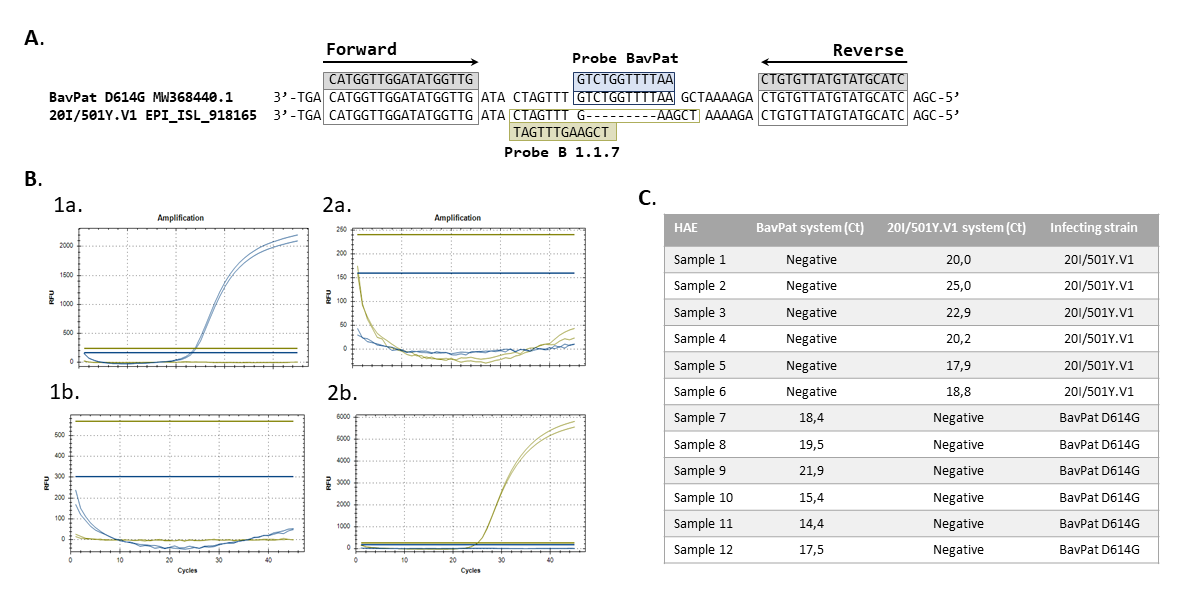


**Supplemental Figure 1:** Design and validation of two specific RT-qPCR systems for BavPat D614G (B.1) and 20I/501Y.V1 (B.1.1.7) strains. A: Detail of the hybridisation position of the common forward and reverse primers. The first nucleotide of the forward primer corresponds to nucleotide 11205 of the reference genome MW368440.1 (gene coding for NSP6). The probe exploiting a 9-nucleotides deletion in the genome of the 20I/501Y.V1 variant in the middle of the amplified sequence achieves the specificity of the system. B: Validation of the systems. Using T7-generated synthetic RNA. Test of specific BavPat system with BavPat (1a.) or 20I/501Y.V1 IVT (1b.). Test of specific 20I/501Y.V1 system with BavPat (2a.) or 20I/501Y.V1 IVT (2b.). No cross amplification was observed between the two systems. C: Results using nucleic acid extracts from HAE supernatant infected with 20I/501Y.V1 variant or BavPat D614G virus. No cross-amplification was observed.
